# Acute kidney injury after heart transplantation: still a shadow after all these years

**DOI:** 10.1590/2175-8239-JBN-2026-E006en

**Published:** 2026-03-09

**Authors:** Renata Mendes, José Hermógenes Rocco Suassuna

**Affiliations:** 1 Universidade do Estado do Rio de Janeiro, Faculdade de Ciências Médicas, Rio de Janeiro, RJ, Brazil.; 2 Universidade Federal do Rio de Janeiro, Rio de Janeiro, RJ, Brazil.; 3 Hospital Pró-Cardíaco, Rio de Janeiro, RJ, Brazil.

In January 1968, Norman Shumway, following the pioneering development of the technique, performed the first successful adult heart transplant in the United States^1^. Despite the surgical success, the patient required peritoneal dialysis from the second postoperative day onward and died due to the progression of complications, most of which can be retrospectively attributed to the initial episode of acute kidney injury (AKI).

After an initial period of enthusiasm, with procedures performed across all continents, including Brazil, the early 1970s brought a reality check. Difficulties related to the still inadequate control of rejection and mortality rates approaching 80% among recipients led to the virtual abandonment of the procedure, with formal moratoriums in some countries^2^. It was the nearly solitary persistence of Shumway and his group that enabled the resurgence of heart transplantation, driven by advances in surgical technique, diagnostics, complication management, and immunosuppression, particularly after the introduction of cyclosporine^2^. However, the expansion of inclusion criteria and the nephrotoxicity of cyclosporine (more intense and severe than in kidney transplantation) brought the loss of renal function to the center of concern^3^.

The kidneys and the heart maintain an interdependent, heterogeneous, and highly complex relationship, which is exacerbated when there is functional impairment of one, the other, or both organs. Interactions along the cardiorenal axis are fundamental to the pathophysiology of heart failure^4^ and persist after heart transplantation^5^. Early series addressing renal dysfunction after transplantation were based predominantly on clinical descriptions, without standardized definitions, and focused on the most severe cases, with imprecise classification of different patterns of renal impairment^6^. Some patients experienced an abrupt decline in renal function; others followed a more indolent course, sometimes starting from preexisting renal disease; and still others showed progressive deterioration of renal function over weeks or months.

Advances in AKI science, with the adoption of consensus diagnostic definitions, have allowed for a more comprehensive understanding of the problem. In parallel, transplant programs progressively expanded acceptance criteria for both donors and recipients, increasingly including patients on prolonged mechanical circulatory support and organs from expanded-criteria donors, thereby intensifying the risk of post-transplant AKI, recently labeled a “rising scourge” in heart transplantation^7^. As a result, despite substantial advances, AKI remains one of the most frequent and severe complications of heart transplantation. In contemporary series, between 25% and 65% of patients require renal replacement therapy (RRT) in the immediate postoperative period^8,9,10,11^.

**Figure 1:**
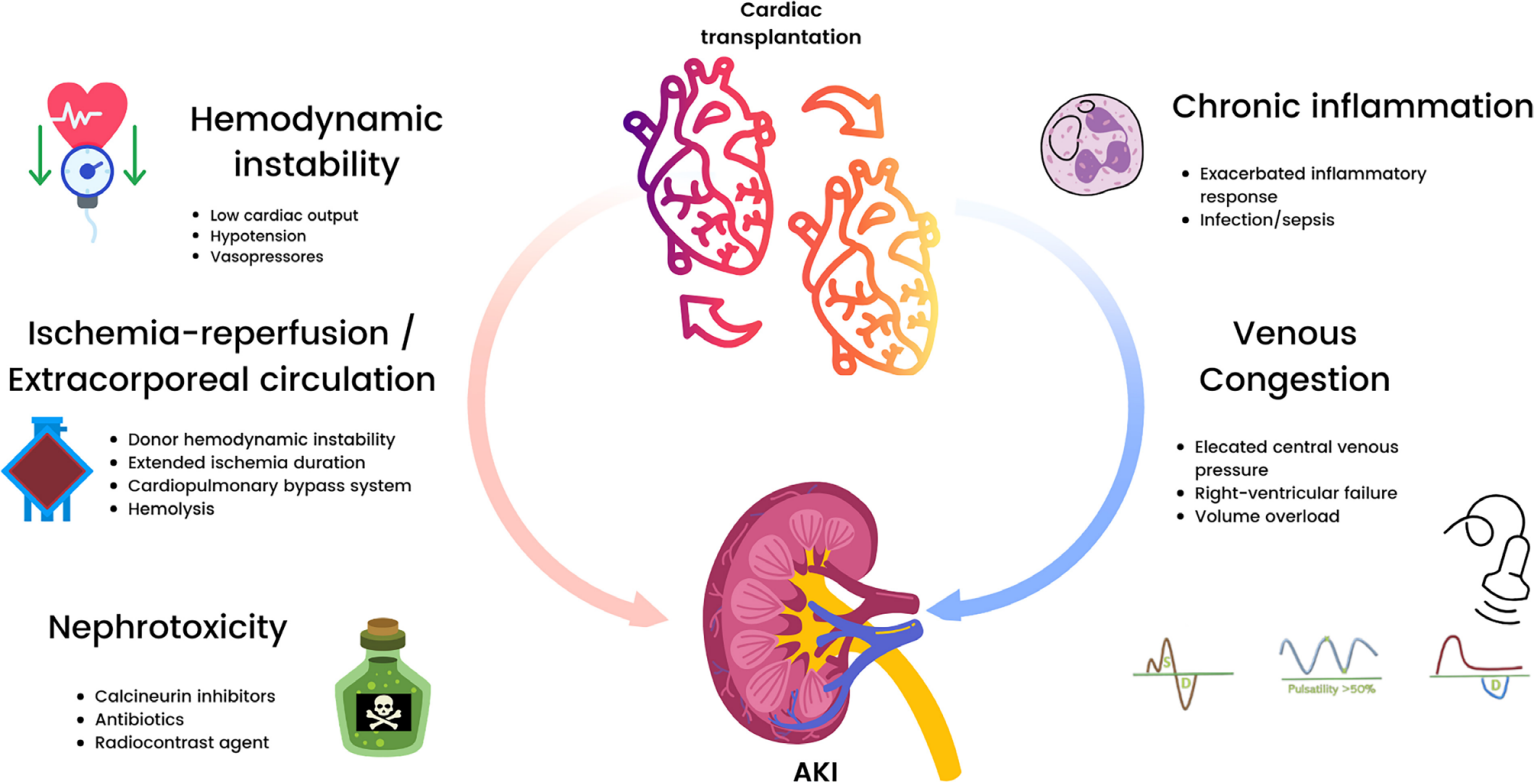
Major risk factors for the development of acute kidney injury in the immediate postoperative period after heart transplantation.

AKI after heart transplantation results from the convergence of hemodynamic instability, ischemia–reperfusion injury, systemic inflammation, exposure to vasopressors, and nephrotoxic immunosuppression^8,9,12^, as summarized in Figure 1. Preexisting renal vulnerability, driven by chronic heart failure, venous congestion, low cardiac output, and repeated hospitalizations, creates a permissive substrate for renal injury even before surgery. Cardiopulmonary bypass contributes to hemolysis, endothelial dysfunction, and the release of inflammatory cytokines, while prolonged ischemia and reperfusion time amplify oxidative and microvascular damage. In the postoperative period, the interaction between graft performance, the need for vasoactive drug support, and renal perfusion is particularly fragile; even modest fluctuations in arterial pressure, effective circulating volume, or systemic congestion may precipitate AKI. Calcineurin inhibitors, still central to most immunosuppressive regimens, add a further nephrotoxic burden.

Abbreviations – AKI: Acute kidney injury; TX: Transplantation.

In this issue of the *Brazilian Journal of Nephrology*, Ferreira et al. applied contemporary diagnostic criteria and reported the experience of an emerging heart transplant program, analyzing 50 patients. Overall, 48% developed AKI across all three KDIGO stages, and 38% required RRT. The report is commendable for highlighting AKI as a significant factor and a determinant of relevant adverse events in the immediate postoperative period (similarly to Shumway’s index patient), particularly by providing data from a local setting. However, the small sample size limits the interpretation of risk factors associated with the outcome of interest. Although the authors performed an extensive univariate analysis, several factors classically associated with the risk of AKI after heart transplantation (Figure 1) were not included or did not reach statistical significance, possibly due to type II error. Subsequently, a logistic regression model was constructed with four variables for 24 events, making overfitting virtually inevitable, even in the presence of an apparently satisfactory R^2^ coefficient, which may compromise the generalizability of their findings^13^.

Despite the magnitude of the problem, there is still a long path ahead to mitigate the risk of AKI after heart transplantation. As recently highlighted, three major barriers persist: the inability to individually predict the risk of AKI before injury is established; the technical impossibility of real-time, integrated monitoring of hemodynamics and renal function during the peri- and postoperative periods; and the lack, to date, of interventions proven to be effective in preventing or treating AKI and its consequences after heart transplantation^14^.

AKI in the setting of heart transplantation is common, multifactorial, and associated with profound consequences. Heart transplantation saves lives, and preserving renal function is an essential condition for ensuring full recovery in these patients. Despite the limitations outlined, the study by Ferreira et al. represents a relevant contribution to addressing this challenge in our setting by providing insights into case profiles, risk variables, and critical points for AKI prevention. Future multicenter studies may refine risk stratification within the national context and eventually inform meta-analyses capable of guiding clinically meaningful practice.
