# Erratum: Acute kidney injury after heart transplantation: still a shadow after all these years

**DOI:** 10.1590/2175-8239-JBN-2026-E006eren

**Published:** 2026-05-15

In the article “Acute kidney injury after heart transplantation: still a shadow after all these years”, with DOI code number: https://doi.org/10.1590/2175-8239-JBN-2026-E006en, published in the journal Brazilian Journal of Nephrology, 48(1):e2026E006, 2026:

In the page 2, where it was written:

“In this issue of the *Brazilian Journal of Nephrology*, Ferreira et al. applied contemporary diagnostic criteria and reported the experience of an emerging heart transplant program, analyzing 50 patients.”

“Subsequently, a logistic regression model was constructed with four variables for 24 events, making overfitting virtually inevitable, even in the presence of an apparently satisfactory R^2^ coefficient, which may compromise the generalizability of their findings^13^.”

“As recently highlighted, three major barriers persist: the inability to individually predict the risk of AKI before injury is established; the technical impossibility of real-time, integrated monitoring of hemodynamics and renal function during the peri- and postoperative periods; and the lack, to date, of interventions proven to be effective in preventing or treating AKI and its consequences after heart transplantation^14^.”

Should read:

“In this issue of the *Brazilian Journal of Nephrology*, Ferreira et al.^13^ applied contemporary diagnostic criteria and reported the experience of an emerging heart transplant program, analyzing 50 patients.”

“Subsequently, a logistic regression model was constructed with four variables for 24 events, making overfitting virtually inevitable, even in the presence of an apparently satisfactory R^2^ coefficient, which may compromise the generalizability of their findings^14^.”

“As recently highlighted, three major barriers persist: the inability to individually predict the risk of AKI before injury is established; the technical impossibility of real-time, integrated monitoring of hemodynamics and renal function during the peri- and postoperative periods; and the lack, to date, of interventions proven to be effective in preventing or treating AKI and its consequences after heart transplantation^15^.”

In the page 3, where it was written:

“13. Austin PC, Steyerberg EW. Events per variable (EPV) and the relative performance of different strategies for estimating the out-of-sample validity of logistic regression models. Stat Methods Med Res. 2017;26(2):796–808. doi: https://doi.org/10.1177/0962280214558972. PubMed PMID: 25411322.

14. Zhu MZL, Marasco SF, Evans RG, Kaye DM, McGiffin DC. Acute kidney injury after heart transplantation: risk stratification is good; risk modification is better-but can we do it? Transplant Direct. 2024;10(6):e1635. doi: https://doi.org/10.1097/TXD.0000000000001635. PubMed PMID: 38769977.”

Should read:

“13. Ferreira MM, Felicio ML, Brito FS, Garcia LR, Ponce D. Acute kidney injury in patients undergoing cardiac transplantation: a cohort study on incidence and risk factors in the first three years of service implementation. Braz. J. Nephrol. 2026;48(1):e20250135.

14. Austin PC, Steyerberg EW. Events per variable (EPV) and the relative performance of different strategies for estimating the out-of-sample validity of logistic regression models. Stat Methods Med Res. 2017;26(2):796–808. doi: https://doi.org/10.1177/0962280214558972. PubMed PMID: 25411322.

15. Zhu MZL, Marasco SF, Evans RG, Kaye DM, McGiffin DC. Acute kidney injury after heart transplantation: risk stratification is good; risk modification is better-but can we do it? Transplant Direct. 2024;10(6):e1635. doi: https://doi.org/10.1097/TXD.0000000000001635. PubMed PMID: 38769977.”

Prof. Dr. Miguel Carlos Riella

Editor-in-chief

