# Supplementary material for: Acute kidney injury after heart transplantation: still a shadow after all these years
Source: J Bras Nefrol. 2026 Mar 9;48(1):e2026E006. doi: 10.1590/2175-8239-JBN-2026-E006en (PMC12970628; doi:10.1590/2175-8239-JBN-2026-E006en)
Supplement: Translation

# Translation: Injúria renal aguda após o transplante cardíaco: ainda uma sombra após todos esses anos

**DOI:** 10.1590/2175-8239-JBN-2026-E006pt

**Authors:** Renata Mendes, José Hermógenes Rocco Suassuna

**Affiliations:** 1 Universidade do Estado do Rio de Janeiro, Faculdade de Ciências Médicas, Rio de Janeiro, RJ, Brasil.; 2 Universidade Federal do Rio de Janeiro, Rio de Janeiro, RJ, Brasil.; 3 Hospital Pró-Cardíaco, Rio de Janeiro, RJ, Brasil.

Em janeiro de 1968, Norman Shumway, após o desenvolvimento pioneiro da técnica, realizou o primeiro transplante cardíaco bem-sucedido em adultos nos Estados Unidos^1^. Apesar do sucesso cirúrgico, o paciente necessitou de diálise peritoneal a partir do segundo dia e faleceu em decorrência da progressão de complicações, a maioria delas atribuíveis, em retrospecto, ao quadro inicial de injúria renal aguda (IRA).

Após uma euforia inicial, com cirurgias realizadas em todos os continentes, inclusive no Brasil, o início dos anos 1970 trouxe um choque de realidade. Dificuldades impostas pelo controle ainda inadequado da rejeição e por uma mortalidade próxima de 80% dos receptores levaram ao virtual abandono do procedimento, com moratórias formais em alguns países^2^. Foi a quase solitária persistência de Shumway e de seu grupo que permitiu o renascimento do transplante cardíaco, impulsionado por avanços na técnica cirúrgica, no diagnóstico, no manejo de complicações e na imunossupressão, especialmente após a introdução da ciclosporina^2^. Contudo, a ampliação dos critérios de inclusão e a toxicidade da ciclosporina (mais intensa e grave do que na transplantação renal) trouxeram a perda da função renal para o epicentro das preocupações^3^.

Os rins e o coração mantêm uma relação interdependente, heterogênea e altamente complexa, que se agrava quando há comprometimento funcional de um, do outro ou de ambos os órgãos. As interações no eixo cardiorrenal são fundamentais na fisiopatologia da insuficiência cardíaca^4^ e persistem após o transplante cardíaco^5^. As séries iniciais sobre disfunção renal após o transplante baseavam-se predominantemente na descrição clínica, sem definições padronizadas, e concentravam-se nos casos mais graves, com classificação imprecisa dos diferentes padrões de disfunção renal^6^. Alguns pacientes cursavam com declínio abrupto da função renal; outros evoluíam de forma mais arrastada, por vezes a partir de nefropatia preexistente; e havia ainda aqueles cuja função renal se deteriorava progressivamente ao longo de semanas ou meses.

O avanço da ciência da IRA, com a adoção de definições diagnósticas consensuais, permitiu uma compreensão mais abrangente do problema. Paralelamente, os programas de transplante expandiram progressivamente os seus critérios de aceitação, tanto para doadores quanto para receptores, muitos destes mantidos sob altas doses de inotrópicos e suporte circulatório mecânico, o que intensificou o risco de IRA no póstransplante, rotulada recentemente como um “flagelo crescente” no transplante cardíaco^7^. Como consequência, mesmo diante de avanços substanciais, a IRA permanece como uma das complicações mais frequentes e graves do transplante cardíaco. Em séries contemporâneas, entre 25% e 65% dos pacientes necessitam de suporte renal artificial (SRA) no período pós-operatório imediato^8,9,10,11^.

A IRA após o transplante cardíaco resulta da convergência de instabilidade hemodinâmica, lesão por isquemia-reperfusão, inflamação sistêmica, exposição a vasopressores e imunossupressão nefrotóxica^8,9,12^, sumarizados na Figura 1. A vulnerabilidade renal pré-existente — impulsionada por insuficiência cardíaca crônica, congestão venosa, baixo débito cardíaco e hospitalizações repetidas — cria um substrato permissivo para injúria renal mesmo antes da cirurgia. Já a circulação extracorpórea contribui para hemólise, disfunção endotelial e liberação de citocinas inflamatórias, enquanto o tempo prolongado de isquemia e reperfusão amplifica os danos oxidativos e microvasculares. No pós-operatório, a interação entre o desempenho do enxerto, as demandas vasoativas e a perfusão renal é particularmente delicada; mesmo flutuações modestas na pressão arterial, no volume circulatório efetivo ou na congestão sistêmica podem precipitar a IRA. Os inibidores da calcineurina, ainda centrais na maioria dos esquemas imunossupressores, impõem um componente adicional de estresse nefrotóxico.

**Figura 1:** Principais fatores de risco para o desenvolvimento da injúria renal aguda no pós-operatório imediato do transplante cardíaco.

Abreviaturas – IRA: Injúria renal aguda; TX: Transplante.

Nesta edição do *Brazilian Journal of Nephrology*, Ferreira et al. utilizaram critérios diagnósticos contemporâneos e relataram a experiência de um programa emergente de transplante cardíaco, analisando 50 pacientes. Globalmente, 48% desenvolveram IRA, considerando os três estágios do critério KDIGO, e 38% necessitaram de SRA. O relato é meritório por destacar a IRA como fator de impacto significativo e determinante de eventos adversos relevantes no pós-operatório imediato (à semelhança do paciente índice de Shumway), especialmente ao trazer dados do nosso meio. Contudo, o número reduzido de pacientes limita a interpretação dos fatores de risco associados ao desfecho de interesse. Embora os autores tenham realizado uma extensa análise univariada, diversos fatores classicamente associados ao risco de IRA após o transplante cardíaco (Figura 1) não foram incluídos ou não atingiram significância estatística, possivelmente por erro do tipo II. Em seguida, foi construído um modelo de regressão logística com quatro variáveis para 24 eventos, tornando praticamente inevitável o sobreajuste (*overfitting*), mesmo diante de um coeficiente R^2^ aparentemente satisfatório, o que pode comprometer a generalização dos seus achados^13^.

Apesar da magnitude do problema, ainda há um longo caminho a percorrer para mitigar o risco de IRA após o transplante cardíaco. Conforme recentemente destacado, três barreiras principais persistem: a incapacidade de prever individualmente o risco de IRA antes que o dano esteja estabelecido; a impossibilidade técnica de monitorização, em tempo real e integrada, da hemodinâmica e da função renal nos períodos peri- e pós-operatórios; e a ausência, até o momento, de intervenções comprovadamente eficazes para prevenir ou tratar a IRA e suas consequências após o transplante cardíaco^14^.

A IRA no cenário do transplante cardíaco é comum, multifatorial e de consequências profundas. O transplante cardíaco salva vidas, e proteger a função renal é condição essencial para garantir a plena recuperação desses pacientes. Apesar das limitações apontadas, o estudo de Ferreira et al. representa uma contribuição relevante para enfrentar esse desafio em nosso meio, ao oferecer informações sobre o perfil de casos, variáveis de risco e pontos críticos para a prevenção da IRA. Estudos multicêntricos futuros poderão aprimorar a estratificação de risco no contexto nacional e, eventualmente, subsidiar meta-análises capazes de orientar condutas clinicamente relevantes.
