# Supplementary material for: Erratum: Acute kidney injury after heart transplantation: still a shadow after all these years
Source: J Bras Nefrol. 2026 May 15;48(2):e2026E006er. doi: 10.1590/2175-8239-JBN-2026-E006eren (PMC13274491; doi:10.1590/2175-8239-JBN-2026-E006eren)
Supplement: Related texts

# Correction: Errata: Injúria renal aguda após o transplante cardíaco: ainda uma sombra após todos esses anos

**DOI:** 10.1590/2175-8239-JBN-2026-E006erpt

No artigo “Injúria renal aguda após o transplante cardíaco: ainda uma sombra após todos esses anos”, com número de DOI: https://doi.org/10.1590/2175-8239-JBN-2026-E006pt, publicado no periódico Brazilian Journal of Nephrology, 48(1):e2026E006, 2026:

Na página 2, onde se lia:

“Nesta edição do *Brazilian Journal of Nephrology*, Ferreira et al. utilizaram critérios diagnósticos contemporâneos e relataram a experiência de um programa emergente de transplante cardíaco, analisando 50 pacientes.”

“Em seguida, foi construído um modelo de regressão logística com quatro variáveis para 24 eventos, tornando praticamente inevitável o sobreajuste (*overfitting*), mesmo diante de um coeficiente R^2^ aparentemente satisfatório, o que pode comprometer a generalização dos seus achados^13^.”

“Conforme recentemente destacado, três barreiras principais persistem: a incapacidade de prever individualmente o risco de IRA antes que o dano esteja estabelecido; a impossibilidade técnica de monitorização, em tempo real e integrada, da hemodinâmica e da função renal nos períodos peri- e pós-operatórios; e a ausência, até o momento, de intervenções comprovadamente eficazes para prevenir ou tratar a IRA e suas consequências após o transplante e a ausência, até o momento, de intervenções comprovadamente eficazes para prevenir ou tratar a IRA e suas consequências após o transplante cardíaco^14^.”

Leia-se:

“Nesta edição do *Brazilian Journal of Nephrology*, Ferreira et al.^13^ utilizaram critérios diagnósticos contemporâneos e relataram a experiência de um programa emergente de transplante cardíaco, analisando 50 pacientes.”

“Em seguida, foi construído um modelo de regressão logística com quatro variáveis para 24 eventos, tornando praticamente inevitável o sobreajuste (*overfitting*), mesmo diante de um coeficiente R^2^ aparentemente satisfatório, o que pode comprometer a generalização dos seus achados^14^.”

“Conforme recentemente destacado, três barreiras principais persistem: a incapacidade de prever individualmente o risco de IRA antes que o dano esteja estabelecido; a impossibilidade técnica de monitorização, em tempo real e integrada, da hemodinâmica e da função renal nos períodos peri- e pós-operatórios; e a ausência, até o momento, de intervenções comprovadamente eficazes para prevenir ou tratar a IRA e suas consequências após o transplante cardíaco^15^.”

Na página 3, onde se lia:

“13. Austin PC, Steyerberg EW. Events per variable (EPV) and the relative performance of different strategies for estimating the out-of-sample validity of logistic regression models. Stat Methods Med Res. 2017;26(2):796–808. doi: https://doi.org/10.1177/0962280214558972. PubMed PMID: 25411322.

14. Zhu MZL, Marasco SF, Evans RG, Kaye DM, McGiffin DC. Acute kidney injury after heart transplantation: risk stratification is good; risk modification is better-but can we do it? Transplant Direct. 2024;10(6):e1635. doi: https://doi.org/10.1097/TXD.0000000000001635. PubMed PMID: 38769977.”

Leia-se:

“13. Ferreira MM, Felicio ML, Brito FS, Garcia LR, Ponce D. Acute kidney injury in patients undergoing cardiac transplantation: a cohort study on incidence and risk factors in the first three years of service implementation. Braz. J. Nephrol. 2026;48(1):e20250135.

14. Austin PC, Steyerberg EW. Events per variable (EPV) and the relative performance of different strategies for estimating the out-of-sample validity of logistic regression models. Stat Methods Med Res. 2017;26(2):796–808. doi: https://doi.org/10.1177/0962280214558972. PubMed PMID: 25411322.

15. Zhu MZL, Marasco SF, Evans RG, Kaye DM, McGiffin DC. Acute kidney injury after heart transplantation: risk stratification is good; risk modification is better-but can we do it? Transplant Direct. 2024;10(6):e1635. doi: https://doi.org/10.1097/TXD.0000000000001635. PubMed PMID: 38769977.”

Prof. Dr. Miguel Carlos Riella

Editor-chefe
